# Altered Brain Functional Activity in Infants with Congenital Bilateral Severe Sensorineural Hearing Loss: A Resting-State Functional MRI Study under Sedation

**DOI:** 10.1155/2017/8986362

**Published:** 2017-02-01

**Authors:** Shuang Xia, TianBin Song, Jing Che, Qiang Li, Chao Chai, Meizhu Zheng, Wen Shen

**Affiliations:** ^1^Department of Radiology, Tianjin First Central Hospital, Tianjin 300192, China; ^2^Department of Nuclear Medicine, Xuanwu Hospital, Capital Medical University, Beijing 100053, China; ^3^Department of Ultrasound, Aerospace Central Hospital, Beijing 100049, China; ^4^School of Law and Politics, Tianjin University of Technology, Tianjin 300384, China; ^5^Department of Radiology, Tianjin Third Central Hospital, Tianjin 300172, China

## Abstract

Early hearing deprivation could affect the development of auditory, language, and vision ability. Insufficient or no stimulation of the auditory cortex during the sensitive periods of plasticity could affect the function of hearing, language, and vision development. Twenty-three infants with congenital severe sensorineural hearing loss (CSSHL) and 17 age and sex matched normal hearing subjects were recruited. The amplitude of low frequency fluctuations (ALFF) and regional homogeneity (ReHo) of the auditory, language, and vision related brain areas were compared between deaf infants and normal subjects. Compared with normal hearing subjects, decreased ALFF and ReHo were observed in auditory and language-related cortex. Increased ALFF and ReHo were observed in vision related cortex, which suggest that hearing and language function were impaired and vision function was enhanced due to the loss of hearing. ALFF of left Brodmann area 45 (BA45) was negatively correlated with deaf duration in infants with CSSHL. ALFF of right BA39 was positively correlated with deaf duration in infants with CSSHL. In conclusion, ALFF and ReHo can reflect the abnormal brain function in language, auditory, and visual information processing in infants with CSSHL. This demonstrates that the development of auditory, language, and vision processing function has been affected by congenital severe sensorineural hearing loss before 4 years of age.

## 1. Introduction

Individuals with congenital sensorineural hearing loss usually have no hearing experiences after birth. Approximately 1‰ to 6‰ newborns suffered from severe to profound sensorineural hearing loss [1–5]. Early hearing deprivation could affect not only language but also cognitive functions, such as decreased execution function, disturbed personality, abnormal social behavior, and delayed decision-making and enhanced visual attention [6–14]. However, the mechanisms of changes in language functions and cognitive functions after hearing loss need further evidence to support.

There is the cross-modal reorganization of auditory-related brain area after long-term hearing deprivation [5, 15]. Functional reorganization of auditory cortex in the patients with hearing loss has been reported by many researchers and the auditory cortex in the deaf could be activated by nonauditory stimulation, such as visual, speech, and vibrotactile stimulation [15–19]. Pathologically, the volume and size of the neurons of cochlear nucleus in deaf animal models are decreased depending on the onset and duration hearing loss [20, 21]. The functional changes of auditory-related brain area remain largely unknown.

Brain development is a gradual process of unfolding of a self-organizing and highly synchronous network from complex interactions between internal and external environment. The studies from both animal models and human children have demonstrated that the maturation of auditory cortex is critical at the first few years of life [22, 23]. Lots of cerebral functions, such as auditory sensory and language, have shown sensitive period. Insufficient or no stimulation of the cortex during the sensitive periods of plasticity could lead to the abnormal function of auditory and language development. Oral speech and language skills would be affected in late-implanted children [24, 25]. The intrinsic mechanisms for language acquisition ability with age in congenital deaf children need to be further investigated.

The advances of fMRI had made it possible to study the intrinsic functional organization of the brain. For example, resting-state fMRI could be used to investigate the spatial-temporal correlations within the functional brain regions during rest, especially in the deaf children who could not cooperate the other kinds of task performance. The amplitude of low frequency fluctuations (ALFF) is one of the parameters to measure the total power in the range of 0.01 and 0.1 Hz which could be an index to reflect the neurophysiological changes in different brain diseases [26–30]. Regional homogeneity (ReHo) reflects the similarities or synchrony of low frequency bold signal fluctuations across the intraregional brain. ReHo, the brain activities as clusters, could change in the different brain disease, such as neuromyelitis optical, stroke, Parkinson, hepatic encephalopathy, and Alzheimer's disease. But until now, little information is available about the changes of intrinsic brain activity in the infants with congenital hearing loss. Here, we hypothesized that there are ALFF and ReHo changes in auditory sensory, language, and vision related brain areas due to congenital hearing loss.

This study aimed to use resting-state functional MRI to study the intrinsic functional changes of brain area due to auditory deprivation in infants with congenital severe sensorineural hearing loss.

## 2. Materials and Methods

### 2.1. Participants

A total of 23 infants with congenital severe sensorineural hearing loss (CSSHL) who did not pass hearing screening using auditory brain stem response (ABR) test at 3 days and 42 days after birth were retrospectively included in our study. The ABR results of all infants showed greater than 90 dB which indicates severe or profound sensorineural hearing loss. The age of deaf infants at MRI examination was from 6 months to 48 months (mean age 24.18 ± 14.00 months). Seventeen age and sex matched normal hearing subjects were recruited. The age of the control group at MRI examination was from 11.2 months to 49 months (mean age 26.35 ± 12.67 months). Excluding criteria include a variety of central nervous system diseases, such as white matter hypoplasia, abnormal neuronal migration, and neuronal skin syndrome, tumor, trauma, infection, epilepsy, and so on. Informed consent was signed by the parents of the infants and all the examinations were approved by the hospital ethics committee.

### 2.2. fMRI Data Acquisition

All the subjects were scanned with MRI at the 3.0T MR scanner (Siemens, Trio) and 16-channel standard quadrature head coil. Before MRI examination, all the infants were given the oral administration of 10% chloral hydrate with an amount of 0.6 mL per kg and the maximum amount of no more than 80 mL.

The detailed MRI sequences include the following: firstly, spin echo (spin echo, SE) and fast spin echo (fast spin echo, FSE) and whole brain T1WI and T2WI were acquired to exclude central nervous system abnormalities. The parameters were as follows: T1WI: TR/TE = 300/2.5 ms, slice thickness = 4 mm, the interlayer spacing = 1.2 mm, matrix = 320 × 320, FOV = 220 × 220 mm, flip angle = 70°, NEX = 1,25 transverse slices covering the whole brain, T2WI: TR/TE = 6000/93 ms, slice thickness = 4 mm, the interlayer spacing = 1.2 mm, matrix = 320 × 320, FOV = 220 × 220 mm, and flip angle = 120°. Secondly, gradient-echo echo planar imaging (GRE-EPI) was scanned and the detailed parameters are TR/TE = 3000 ms/30 ms, FOV = 220 × 220 mm, flip angle = 90°, matrix = 64 × 64, slice thickness = 3 mm, 210 frames, and 38 transverse slices without gap covering the whole brain. Finally, magnetization prepared rapid gradient echo imaging (MP-RAGE) sequences. The parameters were set as follows: TR/TE = 1900/2.53 ms, FOV = 250 × 250 mm, flip angle = 9°, matrix = 256 × 256, slice thickness = 1 mm, and 160 sagittal slices without gap covering the whole brain.

### 2.3. fMRI Data Analysis

The resting-state fMRI data were preprocessed using SPM8 (http://www.fil.ion.ucl.ac.uk/spm) and a pipeline analysis toolbox, REST, and DPARSF (http://www.restfmri.net/) [31–34]. The first ten volumes were discarded. The remaining images were preprocessed using a procedure, which included slice timing correction, head motion correction, T1-weighted image-based spatial normalization to the Montreal Neurological Institute (MNI) space, linear trend removal, and bandpass filtering (0.01–0.08 Hz). All the participants' head motion parameters were less than 3 mm in translation and less than 3 degrees in rotation. We orthogonalized each within-brain voxel's time series with respect to the mean time series from the subject's WM, CSF signals, and the six head motion parameters corresponding to the subject as well as linear and quadratic trends. Average WM and CSF segmentations across all subjects were computed in MNI space. ALFF and ReHo were computed by REST software (http://resting-fmri.sourceforge.net). Finally, the images were smoothed with a Gaussian filter of 6 mm full width at half maximum (FWHM). Template selected was infant template (using 9–15-month-old infant template) provided by the Imaging Research Center (https://irc.cchmc.org/software/infant.php).

### 2.4. Statistics Analysis

Two-sample* t*-test was conducted based on the ALFF and ReHo maps by REST software. Age and gender were selected as covariates. AlphaSim corrected *p* < 0.05 (cluster size > 228 voxels). Correlation analysis was performed to calculate the relationship between ALFF and age in two groups by using SPSS version 19.0 (SPSS Inc. Chicago, IL). Significant difference was set at *p* < 0.05. The value of Cohen *d* was used to describe the effect size (ES).

## 3. Results

### 3.1. ALFF in Infants with CSSHL

ALFF differed significantly between the deaf and hearing group in left Heschl's gyri (BA41) (*p* = 0.0059, ES = 0.94), left superior temporal gyrus (BA22) (*p* = 0.0003, ES = 1.27), left inferior frontal gyrus (BA44) (*p* = 0.0004, ES = 1.27), left inferior frontal gyrus (BA45) (*p* = 0.0000, ES = 2.18), left inferior prefrontal gyrus (BA47) (*p* = 0.0001, ES = 1.40), and left dorsolateral prefrontal cortex (BA46) (*p* = 0.0000, ES = 1.68) in infants with CSSHL (deaf < hearing), which are responsible for auditory, language, and executive function.

**Figure 1:**
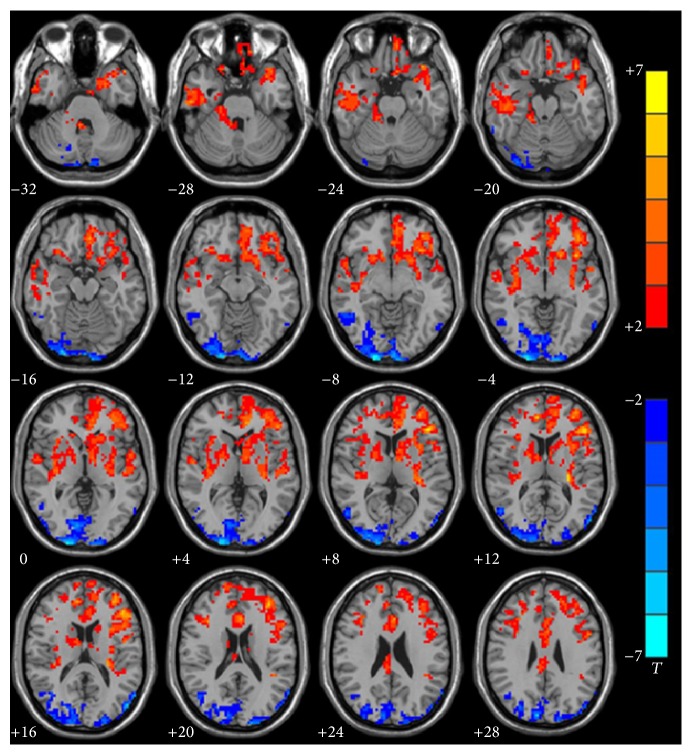
Group analysis of ALFF between infants with CSSHL and healthy controls. Compared with the control group, decreased (red) ALFF was observed in left Heschl's gyri (BA41) (*p* = 0.0059, ES = 0.94), left superior temporal gyrus (BA22) (*p* = 0.0003, ES = 1.27), left inferior frontal gyrus (BA44) (*p* = 0.0004, ES = 1.27), left inferior frontal gyrus (BA45) (*p* = 0.0000, ES = 2.18), left inferior prefrontal gyrus (BA47) (*p* = 0.0001, ES = 1.40), and left dorsolateral prefrontal cortex (BA46) (*p* = 0.0000, ES = 1.68). Increased (blue) ALFF was observed in right occipital lobe and right angular gyrus, which included BA18 (*p* = 0.0022, ES = −1.07), BA19 (*p* = 0.0027, ES = −1.04), BA17 (*p* = 0.0000, ES = −1.46), and BA39 (*p* = 0.0020, ES = −1.08). AlphaSim corrected (*p* < 0.05, cluster size > 228 voxels). The significance level of activity was indicated by the color bar (*T*), increasing as red proceeding to yellow decreasing as blue to cyan.

ALFF value also differed significantly between the deaf and hearing group in right occipital lobe and right angular gyrus, which included right BA18 (*p* = 0.0022, ES = −1.07), right BA19 (*p* = 0.0027, ES = −1.04), right BA17 (*p* = 0.0000, ES = −1.46), and right BA39 (*p* = 0.0020, ES = −1.08) (deaf > hearing), which are responsible for processing visual information (Figure 1, Table 1).

### 3.2. ReHo in Infants with CSSHL

ReHo differed significantly between the deaf and hearing group in left superior temporal gyrus (BA22) (*p* = 0.0117, ES = 0.86), left inferior frontal gyrus (BA45) (*p* = 0.0038, ES = 1.00), left inferior prefrontal gyrus (BA47) (*p* = 0.0016, ES = 1.10), left dorsolateral prefrontal cortex (BA46) (*p* = 0.0076, ES = 0.91), left medial frontal gyrus (BA32) (*p* = 0.003, ES = 1.03), and left temporal polar gyrus (BA38) (*p* = 0.0102, ES = 0.88) (deaf < hearing), which are responsible for auditory processing, language perception, executive function, and behavior and decision-making.

**Figure 2:**
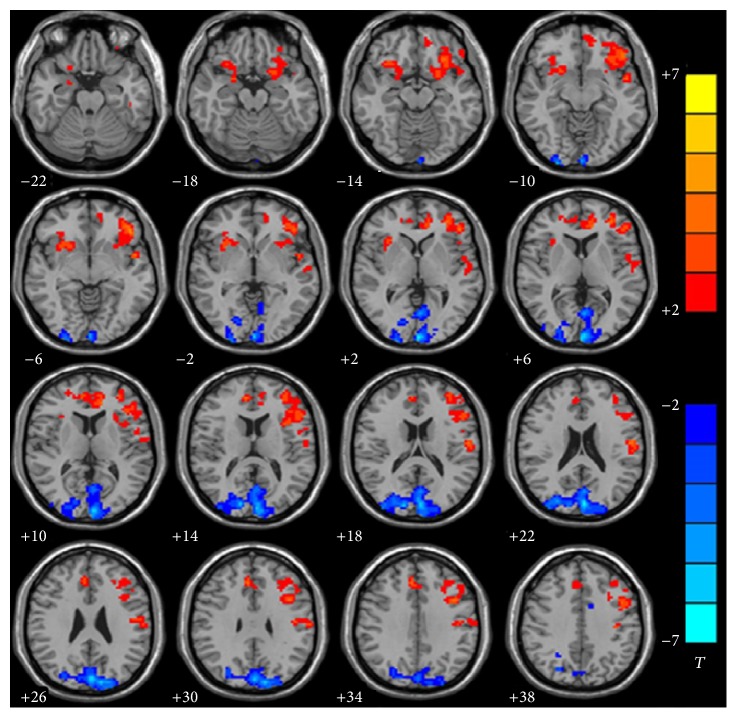
Group analysis of ReHo between infants with CSSHL and healthy controls. Compared with control group, decreased (red) ReHo was observed in left superior temporal gyrus (BA22) (*p* = 0.0117, ES = 0.86), left inferior frontal gyrus (BA45) (*p* = 0.0038, ES = 1.00), left inferior prefrontal gyrus (BA47) (*p* = 0.0016, ES = 1.10), left dorsolateral prefrontal cortex (BA46) (*p* = 0.0076, ES = 0.91), left medial frontal gyrus (BA32) (*p* = 0.0030, ES = 1.03), and left temporal polar gyrus (BA38) (*p* = 0.0102, ES = 0.88). Increased (blue) ReHo was observed in left occipital lobe which included BA18 (*p* = 0.0078, ES = −0.91), BA19 (*p* = 0.0006, ES = −1.20), and BA17 (*p* = 0.0000, ES = −1.41). AlphaSim corrected (*p* < 0.05, cluster size > 228 voxels). The significance level of activity was indicated by the color bar (*T*), increasing as red proceeding to yellow decreasing as blue to cyan.

ReHo value also differed significantly between the deaf and hearing group in left occipital lobe which included BA18 (*p* = 0.0078, ES = −0.91), BA19 (*p* = 0.0006, ES = −1.20), and BA17 (*p* = 0.0000, ES = −1.41) (deaf > hearing), which are responsible for visual information processing (Figure 2 and Table 2).

### 3.3. Relationship between ALFF and Age in Auditory, Language, and Vision Related Brain Areas in Infants with CSSHL

**Figure 3:**
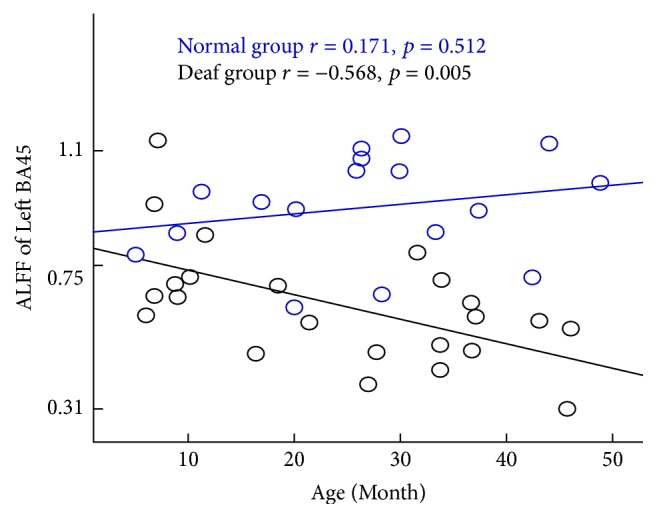
Significant negative correlation between age and the value of ALFF in left BA45 responsible for language production in the deaf group. Deaf group: black (*r* = −0.568, *p* = 0.005). There was a trend of increase along with the increase of age. No significant correlation was found in the control group. Control group: blue (*r* = 0.171, *p* = 0.512).

**Figure 4:**
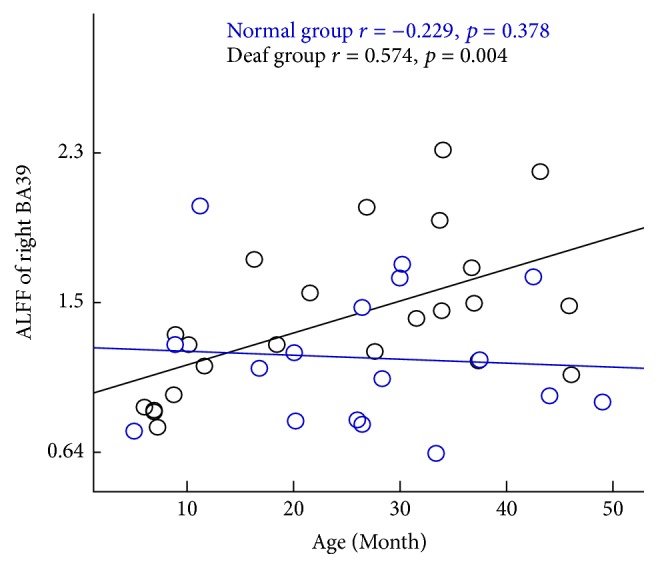
Significant positive correlation between age and the value of ALFF in right BA39 responsible for language processing, spatial orientation, and semantics in the deaf group. Deaf group: black (*r* = 0.574, *p* = 0.004). No significant correlation was observed in the control group. Control group: blue (*r* = −0.229, *p* = 0.378).

The significant increase and decrease ALFF of auditory, language, and visual perception related brain areas were selected as region of interest (ROI) in both groups, which included left BAs 22, 41, 44, 45, 46, and 47 and right BAs 18, 19, and 39. The correlation between ALFF of these ROIs and age was calculated. ALFF of left BA45 was negatively correlated with age and showed a decreasing trend in the deaf group along with age increased (*r* = −0.568, *p* = 0.005), but it showed an increased trend in the control group, though there is no significant correlation (*r* = 0.171, *p* = 0.512) (Figure 3). ALFF of right BA39 was positively correlated with age and showed an increasing trend in deaf group with age (*r* = 0.574, *p* = 0.004). However, there was no significant correlation in control group (*r* = −0.229, *p* = 0.378) (Figure 4).

**Figure 5:**
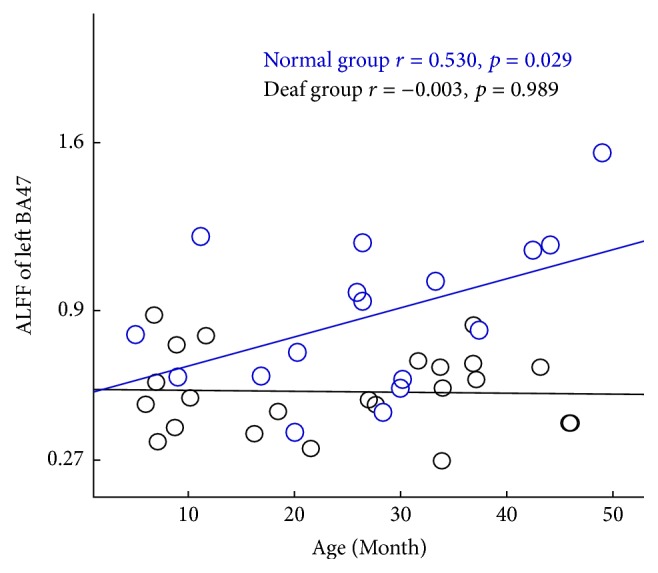
Significant positive correlation between age and the value of ALFF in left BA47 of the control group. Control group: blue (*r* = 0.530, *p* = 0.029). Deaf group: black (*r* = −0.003, *p* = 0.989). No significant correlation was found in the deaf group.

ALFF value of left BA47 was positively correlated with age and showed an increasing trend in the control group (*r* = 0.530, *p* = 0.029), but it showed no significant correlation in the deaf group (*r* = −0.003, *p* = 0.989) (Figure 5). There was no significant correlation between other ROIs and age in both groups (*p* > 0.05). All above results indicated that the function of auditory, language, and vision was changed in infants with CSSHL.

## 4. Discussion

Changes of intrinsic brain organization are essential for exploring infants with the congenital severe sensorineural hearing loss (CSSHL). This is the first study using resting-state fMRI to evaluate the function of auditory and language-related brain areas in infants with CSSHL before four years of age. There were some important findings. ALFF and ReHo values in the deaf infants decreased in auditory, language processing, and executive function related brain areas. ALFF and ReHo values in the deaf infants increased in occipital lobe, which is responsible for visual information processing. Another important finding was that the changes of ALFF of left BA45 were positively correlated with deaf during, and ALFF of right BA39 was negatively correlated with deaf duration in infants with CSSHL. ALFF of left BA47 was also found positively correlated with age in normal subjects again, but the correlation disappeared in infants with CSSHL.

ALFF indirectly reflects the spontaneous neural activity and indicates the functional changes of brain activity [35, 36]. In the present study, ALFF in infants with CSSHL was found decreased in Heschl's gyrus and superior temporal gyrus, inferior frontal lobe, angular gyrus, temporal polar gyrus, and inferior and dorsolateral prefrontal gyrus, which contains BA22, BA41, BA44, BA45, BA46, and BA47, which are responsible for the auditory processing and language perception. The results suggested that the function of auditory and language-related brain cortex was affected due to no sound stimulated. The previous literature also reported that the patients with a profoundly sensorineural hearing loss could have problems in language and learning ability [37–39]. ReHo indicates that topical functional brain area is in a similar activity and abnormal ReHo reflects the desynchronized brain activity [40, 41]. In the present study, ReHo significantly decreased in superior temporal gyrus, inferior frontal gyrus, inferior prefrontal gyrus, dorsolateral prefrontal cortex, and temporal polar gyrus, which contains BA22, BA45, BA47, BA46, BA32, and BA38, in infants with CSSHL. The results also demonstrated that function of auditory and language-related cortex was impaired due to sensorineural hearing loss. It was also reported that glucose hypometabolism was observed in auditory and language-related cortex [42]. Decreased ReHo could reflect the desynchronized blood flow which indicated reduced gray matter concentration [40, 43]. Patients with sensorineural hearing loss have shown lots of brain areas with thinning cortical thickness [44]. The thickness of auditory cortex decreased due to hearing loss and the deafness degree was correlated with the time of deafness onset.

Executive function includes lots of organizational and self-regulatory skills, such as personal and social behavior, decision-making, and emotional and cognitive processing [6, 45] In the present study, ALFF and ReHo were found decreased in inferior prefrontal gyrus (BA47) and dorsolateral prefrontal cortex (BA46), which are responsible for executive function. It suggests that the executive function was abnormal in the infants with CSSHL. Impairment of language function in the deaf children could result in the impaired executive function which could negatively impact the development of language [6].

In deaf infants, not all the executive functions were impaired; some of them are preserved and even enhanced [6, 46, 47]. In the present study, ALFF was found to be increased in right angular gyrus (BA39), which indicated that the spatial orientation and semantic sensation were enhanced compared to the hearing subjects. The study also found that the ability of tracking objects and orientating was enhanced in deaf subjects [12, 13].

The right occipital gyri (BA18, BA19, and BA17) are responsible for visual information processing. In the present study, both ALFF and ReHo values of BA18, BA19, and BA17 increased. It suggests that the vision function had been enhanced due to the hearing loss. Another study also suggested the compensatory enhanced visual process after auditory deprivation, and meanwhile vision cortex is much more sensitive to reorganization or neuroplasticity without auditory input [11, 48]. Behavioral studies had also shown that deaf people have better visual performance [49]. It indicates that vision function is enhanced due to vision cortex reorganization. Neuroplasticity based on the sensory stimulation is present throughout the whole life and the development of the cortex is largely depending on the environment [50]. The sensory cortex and other associated systems, such as cognitive and language, not only interact with each other but also adjust functional characterization according to the stimulation and experience [51].

Interestingly, the significant correlation was found between ALFF of left BA45, right BA39, and deafness duration in deaf infants. ALFF of left BA45 was found to be negatively correlated with the deprivation duration in deaf infants. In contrast, no significant correlation was found between ALFF of left BA45 and age in normal controls although there was an increasing trend along with aging. This demonstrated that the function of left BA45 which is related to language was impaired more seriously along with deaf duration. ALFF of right BA39 was found to be positively correlated with the deprivation duration in deaf infants but with no significant correlation with age in hearing infants. The cortex is responsible for spatial orientation and semantic sensation. This suggests that the function of spatial orientation and semantic sensation was enhanced along prolonged deafness duration. Significant positive correlation was found between ALFF of left BA47 and age in normal infants, but no correlation was found in deaf infants. It may conclude that the function of left BA47 is enhanced along with age increasement in normal infants, but this increased trend disappeared in deaf infants, which suggests that the executive function is the cross-modal plasticity caused by the deprivation of auditory experience.

The primary limitation of this study was the small sample size of infants (hearing loss and hearing group). Additionally, clinical information about the function of auditory and language recovery in deaf infants with cochlear implant was not followed up. The correlation between the recovery and abnormal function of auditory and language-related brain areas in deaf infants' precochlear implant needs to be investigated in the future study.

## 5. Conclusion

In conclusion, ALFF and ReHo reflect the abnormal brain function in language, auditory, and visual information processing in infants with CSSHL before 4 years of age. Congenital severe sensorineural hearing loss affected the development of auditory and language processing function.

## Figures and Tables

**Table 1:** Comparison of ALFF differences between infants with CSSHL and hearing controls.

Region	H	BA	Volume (mm^3^)	Coordinates			Peak	*p*	ES
				*X*	*Y*	*Z*	*T*-value		
Deaf < control									
Heschl's gyrus	L	41	10	−41	−40	18	2.92	0.0059^*∗*^	0.94
Superior temporal gyrus	L	22	16	−56	−17	3	3.93	0.0003^*∗*^	1.27
Inferior frontal gyrus	L	44	20	−52	14	17	3.91	0.0004^*∗*^	1.27
Inferior frontal gyrus	L	45	10	−44	30	10	6.73	0.0000^*∗*^	2.18
Inferior prefrontal gyrus	L	47	62	−42	38	−2	4.33	0.0001^*∗*^	1.40
Dorsolateral prefrontal gyrus	L	46	28	−36	51	17	5.19	0.0000^*∗*^	1.68

Deaf > control									
Occipital gyrus	R	18	278	12	−81	−4	−3.29	0.0022∗	−1.07
Occipital gyrus	R	19	165	27	−68	−4	−3.21	0.0027∗	−1.04
Middle occipital gyrus	R	17	73	11	−87	1	−4.49	0.0000∗	−1.46
Angular gyrus	R	39	31	45	−66	22	−3.32	0.0020∗	−1.08

CSSHL: congenital severe sensorineural hearing loss; H, hemisphere; L, left; R, right; BA, Brodmann's area. AlphaSim corrected (*p* < 0.05, voxel-level cut-off); ES, value of Cohen *d* was used to describe the effect size. ^*∗*^Indicating the significant difference compared with controls.

**Table 2:** Comparison of ReHo differences between infants with CSSHL and hearing controls.

Region	H	BA	Volume (mm^3^)	Coordinates			Peak	*p*	ES
				*X*	*Y*	*Z*	*T*-value		
Deaf < control									
Superior temporal gyrus	L	22	14	−56	−17	3	2.65	0.0117^*∗*^	0.86
Inferior frontal gyrus	L	45	19	−44	−30	10	3.08	0.0038^*∗*^	1.00
Inferior prefrontal gyrus	L	47	74	−42	38	−2	3.40	0.0016^*∗*^	1.10
Dorsolateral prefrontal gyrus	L	46	18	−36	51	17	2.82	0.0076^*∗*^	0.91
Medial frontal gyrus	L	32	45	−11	45	9	3.17	0.0030^*∗*^	1.03
Temporal pole	L	38	52	−30	18	−31	2.70	0.0102^*∗*^	0.88

Deaf > control									
Occipital gyrus	R	18	243	−7	−66	1	−2.81	0.0078^*∗*^	−0.91
Occipital gyrus	R	19	195	−8	−87	27	−3.71	0.0006^*∗*^	−1.20
Medial occipital gyrus	R	17	33	−2	−97	4	−4.35	0.0000^*∗*^	−1.41

CSSHL: congenital severe sensorineural hearing loss; H, hemisphere; L, left; R, right; B, bilateral; BA, Brodmann's area. AlphaSim corrected (*p* < 0.05, voxel-level cut-off); ES, value of Cohen *d* was used to describe the effect size. ^*∗*^Indicating the significant difference compared with controls.
